# Neuropsychiatric symptoms following metal-on-metal implant failure with cobalt and chromium toxicity

**DOI:** 10.1186/s12888-016-1174-1

**Published:** 2017-01-24

**Authors:** Ben Green, Emily Griffiths, Solomon Almond

**Affiliations:** 1 The Institute of Medicine, University of Chester and University Centre Shrewsbury, Bache Hall, Countess Way, Chester, Cheshire CH2 1JR UK; 2 Public Health England, 5 Old Fulwood Road, Sheffield, S10 3TG UK

**Keywords:** Hip implant, Cobalt, Chromium, Depression, Cognitive, Neuropsychiatric, Metal-on-metal

## Abstract

**Background:**

There were at least 31,171 metal-on-metal (MoM) hip implants in the UK between 2003 and 2011. Some of these were subject to failure and widescale recalls and revisions followed.

**Method:**

This is a presentation of ten cases (mean age 60 years) where we evaluated neuropsychiatric morbidity following metal-on-metal hip implant failure and revision. Implants were ASR total hip replacement (acetabular implant, taper sleeve adaptor and unipolar femoral implants) performed between 2005 and 2009. This case series describes, for the first time, neuropsychiatric complications after revision where there has been cobalt and chromium toxicity.

**Results:**

Pre-revision surgery, nine patients had toxic levels of chromium and cobalt (mean level chromium 338 nmol/l, mean cobalt 669.4 nmol/l). Depression assessment showed 9 of 9 respondents fulfilled the BDI criteria for depression and 3 of these were being treated. 7 of 9 patients showing short term memory deficit with mean mini mental state examination score of 24.2. The normal population mean MMSE for this group would be expected to be 28 with <25 indicating possible dementia.

**Conclusions:**

We found neurocognitive and depressive deficits after cobalt and chromium metallosis following MoM implant failure. Larger studies of neurocognitive effects are indicated in this group. There may be implications for public health.

## Background

Metal-on-metal (MoM) hip implants are used in total hip replacement surgeries and hip resurfacing procedures. These implants have both the ball and socket components made of metal. They have been extensively used. The National Registry for Hip Implants in England and Wales recorded 402,051 primary hip implants between 2003 and 2011, of which 31,171 were stemmed metal-on-metal (MoM) hip implants [30]. There have been high failure rates associated with these hip implants: 5-year revision rates in women aged 55 were 8.3% [30]. Regulators including the US Food and Drug Administration, Health Canada and the Therapeutic Goods Administration of Australia have raised concerns about the safety of MoM implants [32]. We looked at the psychiatric impact of these failures in a case series.

After MoM hip resurfacing, higher cobalt and chromium concentrations in blood have been associated with structural changes in visual neurological pathways ([12] study of 29 patients, mean age 59 years). There are reports of metallosis following MoM hip implant failure, but little is known about the specific impacts of chromium and cobalt metallosis from these implants. Cobalt toxicity has been known to follow some arthroplasties since the 1970s [19].

Neurological problems including changes in brain structure and function have been reported following MoM hip resurfacing and higher chromium/cobalt levels [12, 27]. To the best of our knowledge psychiatric symptoms following MoM implants have never previously been described. Herein we present an initial investigation into the neuropsychiatric and cognitive function of patients that had MoM hip implants and required subsequent revision surgery.

Chromium is an essential trace nutrient derived from rocks that exists in various chemical states [20]. Chromium exposure via the respiratory tract is an occupational hazard: acute chromium toxicity symptoms include skin and respiratory tract inflammation, gastrointestinal upset and coma, and, less frequently, renal failure, hepatic impairment, cardiovascular events, haematological effects, teratogenicity and carcinogenicity [5]. Chromium is excreted in bile and urine with little organ retention. In the blood it is divided evenly between plasma and erythrocytes and in the absence of any known exposure, whole blood chromium concentrations in the absence of any known exposure range from 2.0 μg/100 mL to 3.0 μg/100 mL [5].

Cobalt is commonplace in the environment and has over 28 isotopes, some of which are radioactive. Cobalt is a component of cyanocobalamin (vitamin B12) and is widely consumed with an estimated US daily intake of 11 μg/day [6]. Exposure to airborne cobalt causes respiratory symptoms. Animal and human studies have shown toxicity can arise in all major organ systems with exposure to non-radioactive cobalt [1, 6, 26].

Some papers report the physical effects of metallosis following MoM implants [2, 10, 11, 14, 24, 28, 29]. To the best of our knowledge there are no papers specifically describing psychiatric symptoms following MoM implants, but there is a case report indicating neurological problems associated with cobalt and chromium toxicity [27]. Mao and colleagues reported two cases [24]. In one of two published cases of MoM metallosis following ASR hip replacements, Mao et al. [24] showed serum cobalt levels of 410 nmol/l (quoted reference range 0–20) and a serum chromium level of 240 nmol/l (0–100). Concentrations of cobalt and chromium in the joint fluid were 4218 nmol/l and 217 000 nmol/l respectively [9]. Symptoms included appetite and weight loss, depression, cognitive impairment, low energy and metallic taste. Cobalt and chromium levels normalised after revision surgery and symptoms cleared

## Methods

We investigated ten patients who had received MoM hip implants between 2005 and 2009, and who were consecutive referrals for psychiatric opinion following hip implants in the Merseyside area. Patients were not primarily referred because they had an identified affective psychological reaction, or a cognitive deficit, but rather for assessment of their potential psychological response to revision surgery. All ten patients had revision surgery between 2008 and 2012 and were psychiatrically assessed in 2014/15. These implants all involved ASR total hip replacement (acetabular implant, taper sleeve adaptor and unipolar femoral implants).

A consultant psychiatrist (BG) took a comprehensive psychiatric patient history as outlined in standard textbooks (e.g., [18]). The histories were written up and collected into a qualtitative description of typical clinical features in the series e.g., the presence or absence of various psychiatric diagnoses framed according to a standard diagnostic system, i.e., the fifth version of the Diagnostic Statistical Manual - DSM 5 [7].

We assessed participant cognition using the Mini Mental State Examination developed by Folstein et al. [16]. The MMSE gives a maximum score of 30, with the general population having a fairly high score of 28, dipping among those older than 85. It is not used to diagnose psychiatric illness, but can be administered quickly at the bedside by any clinician [15]. A score 25 or less in patients aged over 65 indicates cognitive impairment that should be investigated further [3, 25]. Scores can be affected by age, culture, and education [31].

We assessed participant mood with the Beck Depression Inventory (BDI-II) [8] and by psychiatric interview with a diagnosis of depression according to DSM-5 criteria. Participants were diagnosed with depression if they had a BDI score above 12 [22]. As a benchmark, in one study 500–750 Australian adults with a mean age of 41 had a mean BDI (composite) score of 12.4 [13]. We analysed the sample prevalence of depressive disorder against the prevalence of depression found in Liverpool in the 2001 ODIN study, as Liverpool was the nearest city to the sample population [4].

Our threshold for abnormal neurocognitive function was based on the fact that the mean age of this sample was 60.5 years and that an MMSE of score 25 or less in patients aged over 65 is a general indication of cognitive impairment that should be investigated further [3, 25].

When patients were psychiatrically assessed their mean age was 60.5 years (range 46–71). Six were female and four male. The mean age when implants had been inserted was 52.5 years (range 38–63). The implants were in situ for a mean of 4.44 years (range 2–7).

## Results

### Observed metallosis

We obtained data from standard clinical blood investigations (pre-revision surgery) including levels of cobalt and chromium for all but one of the ten patients. These nine patients had toxic levels of chromium (mean level pre-revision surgery was 338 nmol/l, range 144–664, acceptable levels being <134.5 [23]) and toxic levels of cobalt (mean level pre-revision surgery 669.4 nmol/l, range 184–2470, acceptable levels being <119 [23]).

We only had post revision surgery chromium and cobalt levels for three patients. Of these, two patients had non-toxic chrome and cobalt levels, but one had sub-toxic levels of cobalt (33) and toxic levels of chromium (173).

No patient reported a metallic taste in their mouth.

### Observed mood and cognitive function

We found significantly depressed mood in the nine patients for who responded to the BDI questionnaire (mean BDI score 27.6). This mean score would be consistent with moderately severe depression [8].

We found neurocognitive abnormalities with mean MMSE score of 24.2 across the whole series, with short-term memory deficit in seven out of 10 patients. Other abnormalities included disorientation in place, problems with tests of concentration and word finding difficulties. Two patients had a family history of senile dementia in a first-degree relative. One patient had hypertension and was receiving treatment.

To gain an idea of whether such levels of cognitive function were unexpectedly low in this sample we analysed the sample’s prevalence of neurocognitive deficit against the prevalence of early onset dementia in the population. The best estimate of the prevalence of early onset dementia was between 81 and 113 cases per 100 000 for 45–64-year-olds (i.e., 0.00081–0.00113%) [21]. We observed neurocognitive deficit in seven patients (70%). Using the binomial distribution and an upper bound probability from Lambert et al. of 0.00113, observing seven patients with a deficit from a sample of ten is extremely improbable (*p* < 2.82e-19).

We also analysed the sample prevalence of depressive disorder against the prevalence of depression found in Liverpool, UK in the 2001 ODIN study, which was 7.8% (3.3–17.5 95% CI) amongst adults aged 18–64 years (Ayuso-Mateos 2001). We observed three patients being treated for depression. Using the binomial distribution and a probability from Ayuso-Mateos of 0.078, observing three depressed patients in our sample would only be expected about three times in 100 (*p* < 0.025). All nine patients who completed a BDI survey exceeded the depression threshold of 12, which even if we use the upper confidence interval of 17.5% from Ayuso-Mateos has *p* < 1.54e-07.

The patient with the highest pre-revision surgery levels of chromium (664 nmol/l) and cobalt (2470 nmol/l) had the lowest MMSE score at assessment.

### Patient vignettes

We now describe typical presentations from four patients:

*Ms. X had a right sided ASR total hip replacement - acetabular implant, taper sleeve adaptor and unipolar femoral implant when she was 58 and required revision surgery four years later. She had pre-revision chrome levels of 213 nmol/l and cobalt levels of 249 nmol/l. She was left with persistent anxieties about the need for future surgery and a feeling she must always be cautious about protecting her hip, tearfulness, lability of mood, lowered self esteem – “I used to be very active and now I feel a mess”, guilt about being a burden on her husband, a fear about the effects of ions on her body and poor concentration. She had no cognitive abnormalities and a MMSE score of 30, but had a significantly elevated Beck Depression Inventory Score of 33, after treatment with fluoxetine.*

*Mr. Y had a left sided ASR total hip replacement - acetabular implant, taper sleeve adaptor and unipolar femoral implant when he was 52 and required revision surgery six years later. He had pre-revision chrome levels of 152 nmol/l and cobalt levels of 262 nmol/l. He complained of chronic pain, loss of his occupation, low mood, social withdrawal, poor sleep, poor concentration, short term memory loss noted by relatives, misplacing items round the house, increased irritability, and absent libido. At age 58 he had cognitive abnormalities and a MMSE score of 26, and a significantly elevated Beck Depression Inventory Score of 30 after treatment with dosulepin.*

*Mrs. Z had a right sided ASR total hip replacement - acetabular implant, taper sleeve adaptor and unipolar femoral implant when she was 51 and had revision surgery seven years later. She had pre-revision chrome levels of 326 nmol/l and cobalt levels of 544 nmol/l. She complained of poor sleep with early morning wakening, low mood and emotional lability, social withdrawal, poor appetite, forgetfulness and a tendency to repeat herself reported by relatives, her frustration at being unable to do day to day activities such as cleaning, she felt she was a burden to relatives, and complained of anhedonia. She was disorientated in person, was unable to perform serial sevens and was able to register, but not able to retain any elements of a new name and address on cognitive testing. At age 62 she had cognitive abnormalities and a MMSE score of 17, and a significantly elevated Beck Depression Inventory Score of 38 after treatment with escitalopram.*

*Mrs. A had a right sided ASR total hip replacement - acetabular implant, taper sleeve adaptor and unipolar femoral implant when she was 50 and had revision surgery five years later. She had pre-revision chrome levels of 664 nmol/l and cobalt levels of 2470 nmol/l. She complained of missing her previous occupation, chronic pain, low mood, low self confidence, poor sleep with early morning wakening, poor appetite, forgetfulness with difficulty recalling words, difficulty recalling conversations, social withdrawal, anhedonia, and worry about the need for any further surgery. Her concentration was poor, she was unable to subtract serial sevens or spell the word ‘world’ backwards. At age 57 she had cognitive abnormalities and a MMSE score of 18, and a significantly elevated Beck Depression Inventory Score of 33.*

## Discussion

We present the first case series suggestive of clinically significant depressed mood and neurocognitive impairment following MoM hip failure with concomitant chromium and cobalt toxicity.

Depression might be mediated by psychological mechanisms – fear of the need for further surgery and ongoing problems with pain and mobility being frequently cited concerns. Neurocognitive abnormalities however might be mediated by either static brain damage caused by chromium and cobalt toxicity or could represent a dynamic process, that is an early onset dementia triggered by metallosis. If the latter is the case it might have major, as yet unrecognised, implications for public health.

## Conclusions

We conclude that all clinicians, including those working the fields of orthopaedic, psychiatry and primary care should be aware of the need to assess the neuropsychiatric state of their patients after MoM implant operations. The MMSE and BDI, or similar instruments, would be convenient tools for this. An important corollary of this would be that patients presenting with neuropsychiatric symptoms de novo and who have had orthopaedic implant surgery, should have investigations for chrome and cobalt toxicity.

Other than revision surgery there is no effective adsorption or chelation therapy for chromium and cobalt, and if such therapies could be safely developed, it may avoid the need for further surgery. In the meantime, to preserve neurocognitive function implant removal conceivably should be as soon as possible after toxicity is detected.

There is a possibility of identification of the neuropathological substrate to dementia in such patients [17]. There is to date no neuroimaging study or any autopsy brain tissue analysis on these patients and this would be a future area of research, as would studies looking at the mediating process linking any toxicity with neurocognitive impairment including studies on allergic response and protein accumulation in the brain or direct neurotoxicity

There are additionally potential public health implications for the care needed by many thousands of patients who have potentially suffered MoM related cobalt and chrome toxicity, should progressive cognitive decline be found in this group and the associated requirements for dementia care. This has some and relevance to product liability litigation worldwide. However we believe that any commercial factors should be set aside in the interest of public safety and the bioethical principle of social justice.

## Strengths and limitations

These are novel findings, which are published for the first time, in a small study of sample of patients from a consecutive series of medicolegal referrals, which may have selection bias. A longitudinal study on a larger, randomised cohort is recommended. Small sample sizes can be more susceptible to selection bias and random error, we suggest that further toxicological, neurological, and epidemiological studies be conducted.
